# Considerable interobserver variation calls for unambiguous definitions of thyroid nodule ultrasound characteristics

**DOI:** 10.1530/ETJ-22-0134

**Published:** 2023-03-15

**Authors:** Tamas Solymosi, Laszlo Hegedűs, Steen J Bonnema, Andrea Frasoldati, Laszlo Jambor, Zsolt Karanyi, Gabor L Kovacs, Enrico Papini, Karoly Rucz, Gilles Russ, Endre V Nagy

**Affiliations:** 1 Endocrinology and Metabolism Clinic, Bugat Hospital, Gyöngyös, Hungary; 2 Division of Endocrinology, Department of Medicine, Faculty of Medicine, University of Debrecen, Debrecen, Hungary; 3 Department of Endocrinology, Odense University Hospital, Odense, Denmark; 4 Endocrinology Unit of Arcispedale S Maria Nuova, Reggio Emilia, Italy; 5 Department of Radiology, Faculty of Medicine, University of Debrecen, Debrecen, Hungary; 6 1st Department of Medicine, Flohr Ferenc Hospital, Kistarcsa, Hungary; 7 Regina Apostolorum Hospital in Albano, Rome, Italy; 8 1st Department of Medicine, University of Pecs, Pecs, Hungary; 9 Unité Thyroïde et Tumeurs Endocrines – Pr Leenhardt Hôpital La Pitie Salpetriere, Sorbonne Université, Paris, France

**Keywords:** thyroid nodule, cytology, thyroid cancer, TIRADS, ultrasound

## Abstract

**Objective:**

Thyroid nodule ultrasound characteristics are used as an indication for fine-needle aspiration cytology, usually as the basis for Thyroid Imaging Reporting and Data System (TIRADS) score calculation. Few studies on interobserver variation are available, all of which are based on analysis of preselected still ultrasound images and often lack surgical confirmation.

**Methods:**

After the blinded online evaluation of video recordings of the ultrasound examinations of 47 consecutive malignant and 76 consecutive benign thyroid lesions, 7 experts from 7 thyroid centers answered 17 TIRADS-related questions. Surgical histology was the reference standard. Interobserver variations of each ultrasound characteristic were compared using Gwet’s AC1 inter-rater coefficients; higher values mean better concordance, the maximum being 1.0.

**Results:**

On a scale from 0.0 to 1.0, the Gwet’s AC1 values were 0.34, 0.53, 0.72, and 0.79 for the four most important features in decision-making, i.e. irregular margins, microcalcifications, echogenicity, and extrathyroidal extension, respectively. The concordance in the discrimination between mildly/moderately and very hypoechogenic nodules was 0.17. The smaller the nodule size the better the agreement in echogenicity, and the larger the nodule size the better the agreement on the presence of microcalcifications. Extrathyroidal extension was correctly identified in just 45.8% of the cases.

**Conclusions:**

Examination of video recordings, closely simulating the real-world situation, revealed substantial interobserver variation in the interpretation of each of the four most important ultrasound characteristics. In view of the importance for the management of thyroid nodules, unambiguous and widely accepted definitions of each nodule characteristic are warranted, although it remains to be investigated whether this diminishes observer variation.

## Introduction

For more than three decades, the cornerstones in the clinical management of patients with thyroid nodules have been ultrasound (US) and fine-needle aspiration cytology (FNA) (1, 2, 3). Robust evidence demonstrates that the risk of malignancy (primarily papillary cancer) in thyroid lesions is significantly correlated to the presence of specific US features, which include hypoechogenicity, microcalcifications, taller than wide shape, irregular margins, and extrathyroidal extension (ETE) (4, 5, 6). Several US thyroid nodule risk-classification systems have been proposed by scientific societies (6, 7, 8, 9, 10, 11). These thyroid nodule image reporting and data systems (TIRADS) aim at providing indications for FNA, based on the combined results of the TIRADS malignancy risk scores and nodule size. All of these scoring systems include at least four out of the five suspicious characteristics but are clearly not congruent (12); they handle different microcalcifications, taller-than-wide and taller-than-long shape, and do not define the extent of irregularities which is required to consider a nodule border irregular.

Judgment of the US characteristics of a thyroid nodule can vary widely by the observer (13). The ranges regarding the reported frequency of suspicious characteristics in thyroid cancers vary: 20–100% for hypoechogenicity (4, 6, 14, 15, 16, 17, 18, 19, 20, 21, 22, 23), 14–41% for very hypoechoic nodules (6, 19, 20), 13–56% for microcalcifications (14, 18, 19, 20, 21, 22, 23), and 13–48% for irregular margins (6, 14, 19, 20, 21). Such between-study differences in the prevalence of US characteristics may be explained by differences in (i) the ratio of benign and malignant cases in the study cohort, (ii) the prevalence of follicular or medullary cancer in the given series, due to the ambiguous US features that may be presented by these less frequent types of cancer, (iii) histopathological confirmation and interpretation, (iv) iodine intake, (v) a bias toward smaller and therefore easier to analyze lesions, (vi) the US equipment used, or (vii) image interpretation.

The aim of the current study was to analyze video recordings, rather than still images of histologically verified lesions, in order to determine the inter- and intra-observer variations of nodule characteristics in predicting thyroid cancer. To this end, seven highly experienced investigators from as many centers participated in the analysis. Instead of the traditional kappa-values and percentage agreement, the more novel statistical analyses, Gwet’s agreement coefficient (Gwet’s AC1) was applied (24).

## Materials and methods

### Patients and video records

Between January 2014 and December 2016, the US examinations of 16,407 consecutive patients were video-recorded using a pre-specified protocol (see Supplementary text, see section on supplementary materials given at the end of this article) and archived at the Thyroid Clinic of the Bugat Pal Hospital (Gyöngyös, Hungary) as part of the institutional routine record keeping. A Philips CX 50 US machine equipped with a 12-5 MHz linear transducer was used for thyroid US. Statistical power calculations have shown that a minimum of 102 cases, including at least 39 malignant cases, were required. We added 20% to the calculated number to ensure ample power. In total, 709 cases had surgery for nodular goiter. From this chronological list of patients, the starting point was chosen at random, and the US video records of 47 and 76 patients with malignant and benign final histology, respectively, were used. The indication for surgery was based on cytology in 79 patients (Bethesda IV in 19 patients, Bethesda V in 32 patients, and Bethesda VI in 28 patients), symptoms and/or signs of compression caused by the goiter in 35 cases, an autonomously functioning nodule causing hyperthyroidism in 5 patients, and patients’ wish in 4 cases. Final diagnoses were, in all cases, obtained by histological examination of the surgical samples. Relevant patient data appear in Table 1.

**Table 1:** Postoperative thyroid nodule histology and tumor stage.

Histology	*n*	Tumor status^a^	Tumor stage^a^	Male/female	Mean age (range)
Benign	76	n/a	n/a	18/58	51.1 (24–75)
No nodule	4	n/a	n/a	0/4	49.3 (38–60)
Hyperplastic nodule	43	n/a	n/a	10/33	54.8 (30–75)
Adenoma	29	n/a	n/a	8/21	45.9 (24–67)
Malignant	47	T1 *n* = 25 T2 *n* = 8 T3 *n* = 2 T4 *n* = 11 n.a. = 1		13/34	44.6 (18–89)
Papillary carcinoma	37	T1 *n* = 22 T2 *n* = 7 T3 *n* = 1 T4 *n* = 7	Stage 1 *n* = 33 Stage 2 *n* = 2 Stage 3 *n* = 1 Stage 4 *n* = 1	11/26	42.5 (18–67)
Follicular carcinoma	3	T1 *n* = 2 T3 *n* = 1	Stage 1 *n* = 3	0/3	51 (21–53)
Poorly differentiated cancer	1	T1 *n* = 1	Stage 1 *n* = 1	0/1	42
Anaplastic carcinoma	2	T4 *n* = 2	Stage 4 *n* = 2	2/0	69.5 (57–82)
Medullary carcinoma	2	T2 *n* = 1 T4 *n* = 1	Stage 2I *n* = 1 Stage 4V *n* = 1	0/2	65.5 (32–89)
B-cell lymphoma	1	n/a	Stage 2 *n* = 1	0/1	49
Parathyroid carcinoma	1	T4 4 *n* = 1	Stage 4 *n* = 1	0/1	54
Total	123				

^a^TNM classification of malignant tumors (50).

n/a, not applicable.

Representative parts of each US video recording were presented to seven investigators (see later), who were blinded to the outcome data. The video recordings are available for the reader (http://thyrosite.com/case_studies/section08/consecutively_operated.php).

Written consent has been obtained from each patient after a full explanation of the purpose and nature of all procedures used. The study was approved by the Research Ethics Committee of Bugat Hospital, Gyöngyös, Hungary.

### Evaluation phase

The expert evaluations were performed online using a website developed for this purpose. Seven investigators, from different thyroid centers in four European countries, with at least 15 years of experience in thyroid US (SB, AF, LJ, GK, EP, KR, and GR) analyzed the US video recordings of the 123 histologically verified thyroid lesions. The investigators were aware that all lesions had been surgically removed but blinded to the final histopathology and the benign-to-malignant ratio of the series of nodules under examination (25). In order to reproduce a setting similar to the real world, a short summary of the pre-US clinical data, including thyroid hormone and antibody levels, was provided to the investigators.

During the training phase, 1 month before the study started, ten nodules from ten patients (not included in the study of the 123 cases) were analyzed by the seven investigators. The aim was for the investigators to become acquainted with the study methodology and resolve any questions before the launch of the study case series. The steering committee (LH, TS, and EVN) resolved any issue raised by the US investigators. No further communication among investigators or with the steering committee was allowed.

The 123 cases were presented one by one, in random order, to each investigator. The transducer orientation above the upper, middle, or lower as well as the medial or lateral lobe region was indicated. A still image of the whole gland was also included and the position of the nodule to be studied was shown. The videos (median duration 43 s, range 20 to 73 s) allowed slow-motion assessment, repeat evaluation, and image-freezing, without time constraints. After the analysis of each video, investigators answered the questions in the electronic Case Report Form (CRF). The questions pertained to various US features, including four widely accepted suspicious US characteristics in relation to nodule echogenicity, microcalcifications, irregular borders, and ETE (Supplementary Table 1). To simulate a real-life evaluation, investigators could not modify their answers or re-review the video recordings once the ‘patient completed’ button had been activated. Four weeks were allowed for the completion of the 123 cases.

Eight weeks after completing the evaluation of the 123 nodules (first run), the investigators were requested to repeat the full analysis (second run), which all accepted. The video recordings were presented in a revised computer-generated random order, different from the first run.

### Statistical analysis

We used Gwet’s Agreement Coefficient (AC1) values to analyze inter- and intra-observer concordance and reliability (24). For comparability with other studies, we also calculated the traditional (Cohen’s and Fleiss’) Kappa values. However, Kappa counting provides some ‘meaningless’ values (26). Others also found that interrater variability is more accurately described by percentage agreement than Kappa, if raters are well trained and little guessing is likely to exist during the evaluation process (27). Therefore, Gwet’s AC1 was considered to be the appropriate statistical method for agreement studies. In contrast to Kappa, the Gwet’s AC1 provides a more realistic estimate for the chance effect, is more stable against marginal probabilities, and can handle ordinal scales well (28). We used the following categories for the description of the degree of agreement: poor, fair, moderate, good, and very good, corresponding to Gwet’s AC1 values ≤0.20, 0.21–0.40, 0.41–0.60, 0.61–0.80, and 0.81–1.00, respectively (29).

For testing the influence of nodule size (large, middle, or small) on the agreement among investigators, tertiles of each of the histologically proven entities of (i) hyperplastic nodules, (ii) adenomas, and (iii) papillary carcinomas, each containing one-third of the respective pathology, were created. The answers by the investigators to the following five questions were analyzed separately in each size group: presence of microcalcifications; irregular margins; ETE; iso-, hyper-, or hypoechogenic appearance; and if hypoechogenic whether minimally, moderately, or very hypoechogenic. Chi-square tests were used for comparisons.

While testing the suspicious characteristics for predicting malignancy, the sum of yes and no answers was compared to the final histopathology. Sensitivities, specificities, and the 95% confidence intervals (95% CI) were calculated by the package ‘epiR’ in R version 1.0–2 (30).

## Results

### Microcalcifications and punctate echogenic foci

Moderate (AC1 = 0.53) and fair (AC1 = 0.39) interobserver agreements were found for microcalcifications (CRF question 5) and for punctate echogenic foci (CRF question 4), respectively (Table 2). The percentage of nodules in which punctate echogenic foci were deemed to be present by the investigators ranged from 39.4 to 93.5%, while the range for unequivocal microcalcifications was 9.3 to 50.4% (Table 3).

**Table 2:** The concordance between the seven investigators in the judgment of ultrasound characteristics, listed according to Gwet’s AC1, starting with the lowest concordance. The numbers in the characteristic/property/feature column identify the corresponding question answered by the investigators for each nodule. For comparison purposes, kappa is also shown as earlier studies used kappa values.

Characteristic/property/feature	No. of nodules analyzed	Interobserver mean (95% CI)		Intraobserver mean (95% CI)	
		Gwet’s AC1 value	Fleiss kappa	Gwet’s AC1 value	Cohen’s kappa
6. Uncertain hyperechogenic spots	123	0.12 (0.05–0.19)	0.05 (0.00–0.10)	0.48 (0.42–0.54)	0.48 (0.42–0.53)
13/B. Mild/moderately vs very hypoechogenic nodule^a^	39	0.17 (0.06–0.27)	0.07 (−0.03–0.16)	0.63 (0.56–0.70)	0.57 (0.49–0.64)
12/B. Does a partially cystic nodule have an eccentric solid part?^b^	9	0.28 (−0.16–0.72)	0.26 (−0.09–0.60)	0.78 (0.70–0.86)	0.71 (0.61–0.81)
14. Irregular margins	123	0.34 (0.26–0.42)	0.18 (0.14–0.24)	0.62 (0.57–0.67)	0.51 (0.47–0.56)
4. Punctate echogenic foci	123	0.39 (0.29–0.49)	0.27 (0.21–0.33)	0.68 (0.63–0.72)	0.52 (0.48–0.57)
3. Back wall cystic figures	123	0.48 (0.38–0.57)	0.11 (0.07–0.16)	0.74 (0.70–0.78)	0.49 (0.43–0.55)
5. Microcalcification	123	0.53 (0.43–0.63)	0.29 (0.22–0.36)	0.73 (0.69–0.78)	0.59 (0.53–0.65)
2. Comet-tail artifact	123	0.62 (0.53–0.70)	0.23 (0.15–0.30)	0.78 (0.74–0.81)	0.48 (0.42–0.54)
12/A Is a nodule partially cystic?	123	0.63 (0.54–0.72)	0.50 (0.41–0.59)	0.77 (0.73–0.81)	0.71 (0.66–0.76)
13. Echogenicity of a nodule	123	0.72 (0.68–0.76)	0.24 (0.19–0.29)	0.81 (0.79–0.84)	0.53 (0.49–0.58)
13/A hyper/isoechogenic vs hypoechogenic nodule^c^	74	0.73 (0.63–0.83)	0.43 (0.29–0.57)	0.79 (0.75–0.83)	0.67 (0.61–0.74)
12. Partially cystic nodule	123	0.79 (0.73–0.85)	0.40 (0.34–0.48)	0.86 (0.83–0.88)	0.67 (0.62–0.72)
15. Extrathyroidal extension	123	0.79 (0.73–0.85)	0.28 (0.15–0.41)	0.87 (0.85–0.90)	0.56 (0.48–0.64)
7. Coarse calcification	123	0.80 (0.73–0.87)	0.46 (0.34–0.57)	0.87 (0.85–0.90)	0.65 (0.59–0.71)
11. Solid vs cystic nodule	123	0.84 (0.79–0.89)	0.50 (0.44–0.58)	0.90 (0.87–0.92)	0.66 (0.61–0.70)
8. Central intranodular coarse calcification	123	0.86 (0.81–0.91)	0.40 (0.28–0.52)	0.92 (0.89–0.94)	0.62 (0.55–0.69)
10. Peripheral (rim) calcification	123	0.92 (0.89–0.95)	0.21 (0.13–0.29)	0.95 (0.94–0.96)	0.45 (0.38–0.52)
1. Nodule or not nodule	123	0.94 (0.91–0.97)	0.12 (0.02–0.22)	0.97 (0.96–0.98)	0.62 (0.47–0.77)
9. Isolated macrocalcification occupying the entire nodule	123	0.98 (0.97–0.99)	−0.01 (−0.02–0.00)	0.98 (0.97–0.99)	0.26 (0.09–0.43)

^a^Calculation 13/B was performed for responses to Question 13 which found any degree of hypoechogenicity; ^b^Calculation 12/B was performed for ‘yes’ responses to Question 12; ^c^Calculation 13/A was performed using the respective responses to Question 13.

**Table 3:** The percentage of the nodules examined (*n* = 123) in which the seven investigators deemed the given characteristic to be present (all values are given in %; mean, s.d., minimum, and maximum of the individual % values of the seven investigators).

Feature	Answer	Mean	s.d.	Minimum	Maximum
Punctate echogenic foci					
	Present	53	19.1	39.4	93.5
	Probably present	33.3	14.3	6.1	48.0
	Probably absent	8.7	6.5	0.4	18.3
	Absent	5.1	4.6	0	13.8
Microcalcification					
	Present	27.1	14.4	9.3	50.4
	Absent	72.9	14.4	49.6	90.7
Extrathyroidal extension					
	Present	12.8	5.9	4.1	19.9
	Absent	87.2	5.9	80.1	95.9
Margins					
	Smooth	45.2	16.9	22	63.4
	Ill-defined	26.1	11.2	7.7	39.8
	Irregular	20.8	8.9	11.4	34.1
	Cannot be determined	7.8	9.1	0	18.3
Echogenicity					
	Iso-/hyperechogenic	24.2	12.9	8.1	41.1
	Mildly/moderately hypoechogenic	43.7	12.8	28.5	60.2
	Very hypoechogenic	21.5	11.6	7.7	37.4
	Anechoic	2.7	1.5	0.8	5.3
	Cannot be determined	7.8	11.1	0	28.0
Echogenicity (iso-/hyperechogenic and hypoechogenic nodules only)					
	Iso-/hyperechogenic	26.9	13.3	8.2	41.9
	Hypoechogenic	73.1	13.3	58.1	91.8
Comet-tail artifact					
	Present	18.0	7.4	7.3	27.2
	Absent	72.6	13.7	46.7	84.1
	Uncertain	9.3	9.7	0	27.2
Back-wall cystic figure					
	Present	21.1	10.9	3.7	35.8
	Absent	70.4	12.6	51.6	90.7
	Uncertain	8.5	8.0	0	24.0
Macrocalcification					
	Present	17.8	7.0	11.4	27.2
	Absent	79.6	6.5	70.7	87.8
	Uncertain	2.7	2.1	0	6.5
Is the lesion a nodule?					
	Yes	96.5	4.0	87.8	99.6
	No	3.5	4.0	0.4	12.2

### Echogenicity of the nodule

Good (AC1 = 0.72) and very good (AC1 = 0.81) interobserver and intraobserver agreements, respectively, were found for overall general. Both the interobserver (AC1 = 0.73) and intraobserver agreements (AC1 = 0.67) proved to be good in the distinction between iso/hyperechogenic vs hypoechogenic nodules. On the other hand, the agreements were poor (AC1 = 0.17) and good (AC1 = 0.63) for the interobserver and intraobserver variation, respectively, in the differentiation between minimally/moderately and very hypoechogenic nodules (CRF question 13) (Table 2).

The percentage of nodules which were deemed to be iso/hyperechogenic by the investigators ranged from 8.1 to 41.1%. For the level of hypoechogenicity, 28.5 to 60.2% and 7.7 to 37.4% of the nodules were found to be mildly/moderately or very hypoechogenic, respectively.

### Margins of the nodule

Fair (AC1 = 0.34) and good (AC1 = 0.62) agreements were found for interobserver and intraobserver variations, respectively (CRF question 14) (Table 2). The percentage of nodules in which irregular margins were deemed to be present by the investigators ranged from 11.4 to 34.1% (Table 3).

### Extrathyroidal extension

Regarding the presence of ETE, good (AC1 = 0.79) and very good (AC1 = 0.87) agreements were found for interobserver and intraobserver variation, respectively (CRF question 15) (Table 2). The percentage of nodules in which the investigators deemed ETE to be present ranged from 4.1 to 19.9% (Table 3).

A biological standard, namely pathology, exists for this US characteristic. Thus, we compared pathology results with US findings. ETE was correctly identified by US in only 45.8% of the cases. We analyzed the sensitivity of detecting ETE in relation to nodule size tertile; the values for small nodules (maximal diameter <17 mm), middle-size nodules (diameter between 17 and 29 mm), and large nodules (maximal diameter ≥30 mm) were 78.6, 44.6, and 31.7%, respectively ( = 0.0001).

### The presence/absence of a nodule: was there a nodule at all?

This is another characteristic for which pathology defines the biological standard. The investigators provided a correct answer in 4 of 28 (14.3%) cases in which no nodule was found on histopathology. However, both the interobserver (AC1 = 0.94) and intraobserver agreements proved to be very good (AC1 = 0.97) for the presence/absence of a nodule (CRF question 1) (Table 2).

### Other characteristics

The interobserver agreement was good (AC1 = 0.80) in various subtypes of calcifications (CRF question 7 to 10) and in the composition of nodules (CRF question 11). The agreement was good (AC1 = 0.62) and moderate (AC1 = 0.48) in the judgment of comet-tail artifacts (CRF question 2) and back wall cystic figures (CRF question 3), respectively.

### The influence of the size of the nodule on the interobserver agreement

**Figure 1:**
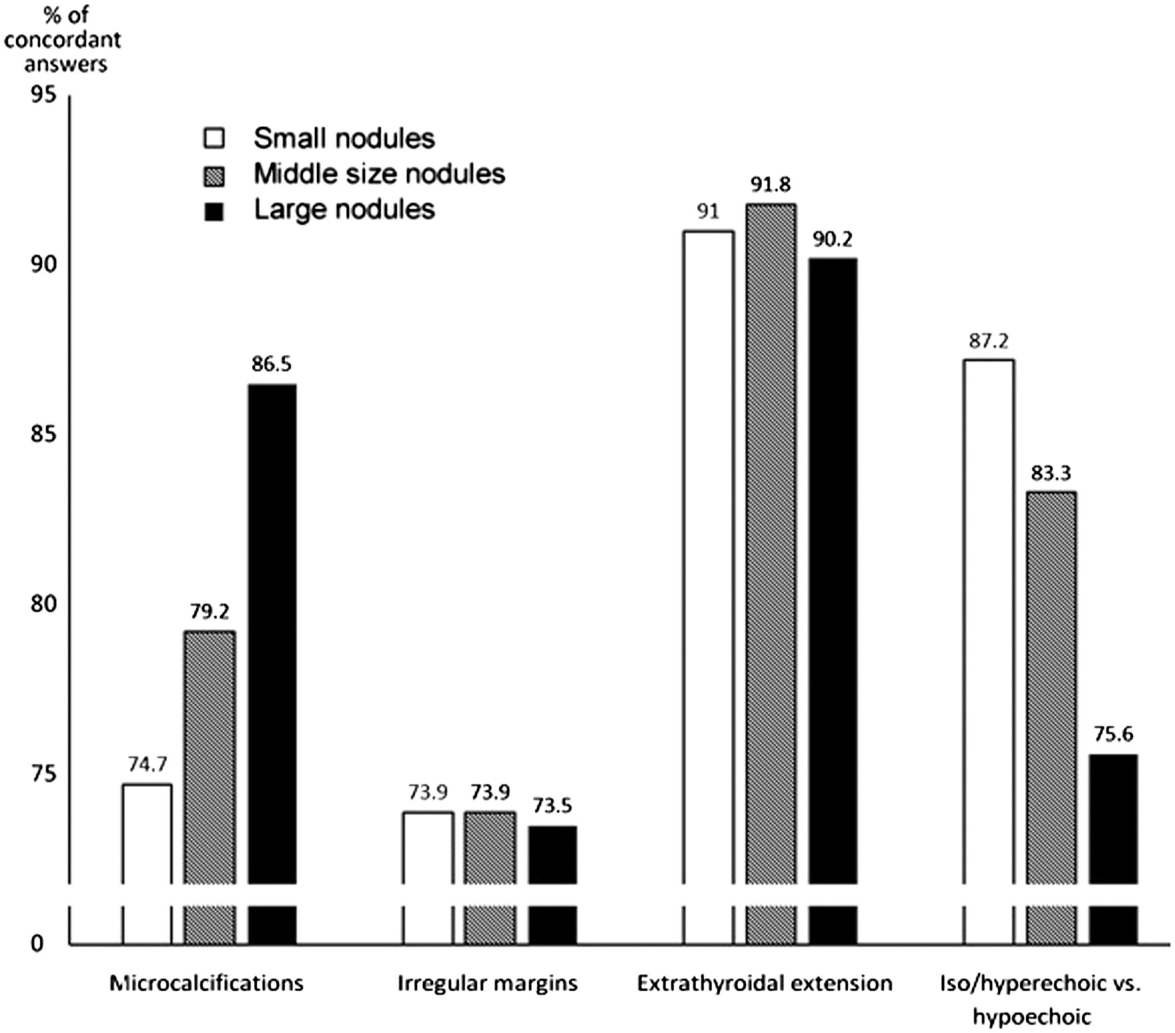
The influence of nodule size on interobserver agreement. Nodules are grouped according to nodule size tertiles (small nodules with a maximal diameter <17 mm, middle-size nodules 17–29 mm, and large nodules ≥30 mm).

For microcalcifications, the larger the nodule size the better the agreement (*P* = 0.004, chi-square = 11.0). When differentiating between iso/hyperechogenicity and hypoechogenicity, agreement was better for the small nodules (*P* = 0.005, chi-square = 10.5). In contrast, size did not influence interobserver agreement in the discrimination between minimally/moderately and very hypoechogenic nodules, irregular margins, or ETE (Fig. 1).

### The diagnostic value of suspicious characteristics in predicting thyroid cancer

The 47 malignant and 76 benign cases resulted in 329 and 532 answers, respectively, from the seven investigators. The diagnostic sensitivity of microcalcifications in predicting malignancy was 42.2% (139 out of 329 answers), while the specificity was 82.9% (441 out of 532 answers). For ‘very hypoechogenic’ echogenicity and all degrees of hypoechogenicity, respectively, the diagnostic sensitivity in predicting malignancy was 37.4% (123 out of 329 answers) and 80.2% (264/329), while the specificity was 87.8% (467 out of 532 answers) and 46.6% (248 out of 532 answers), respectively. The diagnostic sensitivity of irregular margins in predicting malignancy proved to be 37.4% (123 out of 329 answers), while the specificity was 88.3% (470 out of 532 answers).

Eight cases showed ETE, while 115 cases did not, which resulted in 56 (ETE present) and 805 (ETE absent) answers from the seven investigators. The diagnostic sensitivity of ETE in predicting malignancy was 26.8% (15 out of 56 answers), while the specificity was 95.9% (772 out of 805 answers) (Table 4).

**Table 4:** The diagnostic sensitivity and specificity of suspicious ultrasound characteristics for predicting thyroid cancer. Comparison with the previously published data (25, 37, 38, 39, 40, 41, 42, 43, 44, 45, 46, 47, 48, 49).

	Sensitivity (%)		Specificity (%)	
	Present study	Literature – median (range)	Present study	Literature – median (range)
Hypoechoic	80.2% (264/329)	65.4% (20.0–100.0)	46.6% (248/532)	64.6% (43.4–92.0)
Very hypoechoic	37.4% (123/329)	17.4% (14.2–41.4)	87.8% (467/532)	97.1% (92.2–97.1)
Microcalcifications	42.2% (139/329)	36.9% (12.5–55.8)	82.9% (441/532)	91.6% (12.1–98.0)
Irregular margins	37.4% (123/329)	44.2% (13.0–48.3)	88.3% (470/532)	90.0% (69.1–98.4)
Extrathyroidal extension	26.7% (88/329)	20.8% (one study)	95.9% (510/532)	97.5% (one study)

## Discussion

This is the first study using US video recordings of consecutively operated patients for comparison of the evaluations of nodule characteristics. Employing highly experienced investigators the diagnostic value of individual US features was analyzed. Our study design minimized factors which might have caused bias in other studies. Thus, large and difficult-to-examine nodules were not excluded, the investigators did not have a common educational background, and the use of real-time videos rather than one or a few preselected still images simulated the real-world situation. Furthermore, we used an international and diverse group of investigators, because in single-institution studies, investigators are likely to interpret US signs more uniformly.

**Figure 2:**
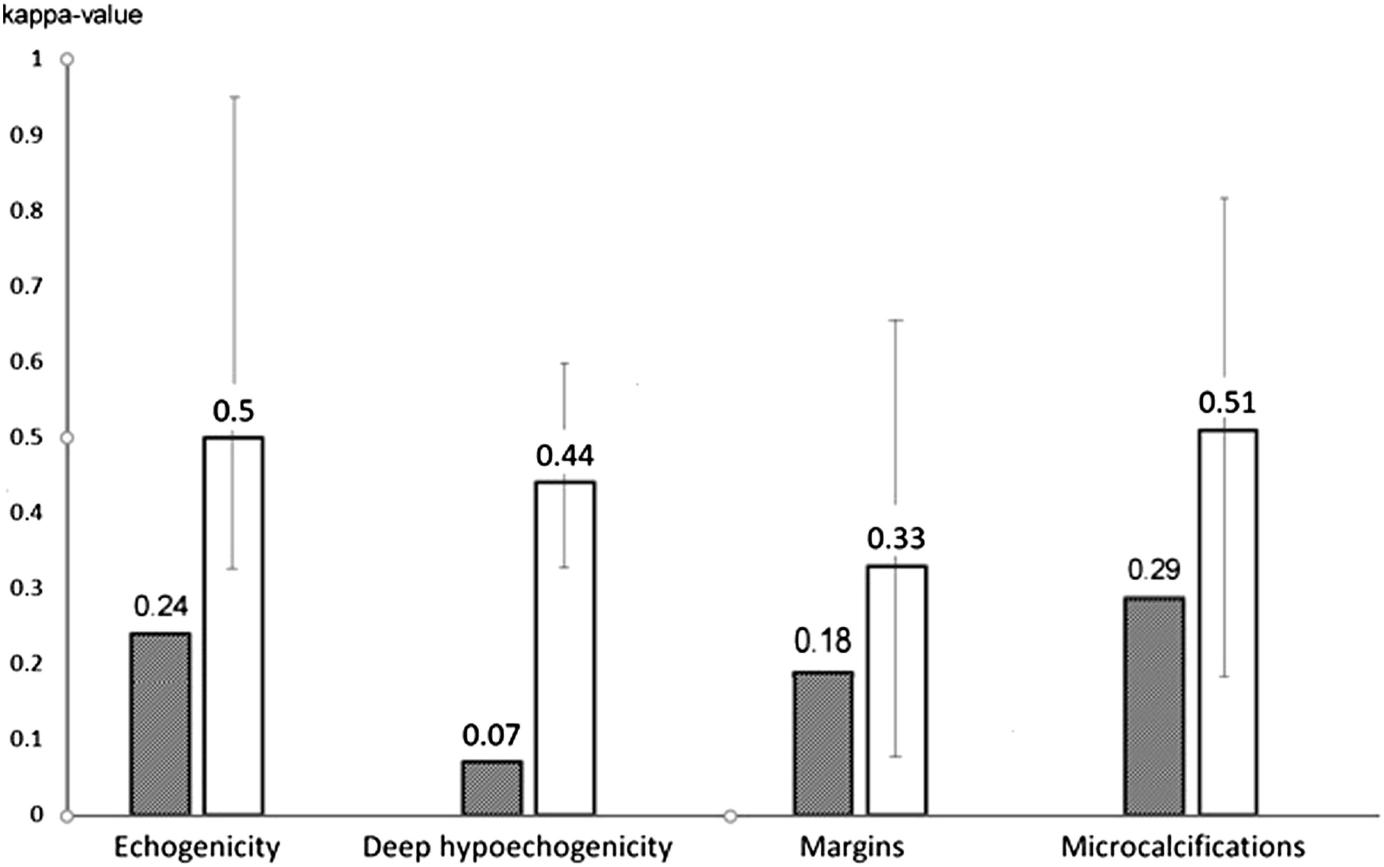
Interobserver variation in the interpretation of certain ultrasound characteristics. For comparison purposes, we calculated Kappa-s, as earlier studies used Kappa values (4, 6, 14, 15, 16, 17, 18, 19, 20, 21, 22, 23). Gray bars: present study; white bars: median of literature data. Error bars represent ranges.

**Figure 3:**
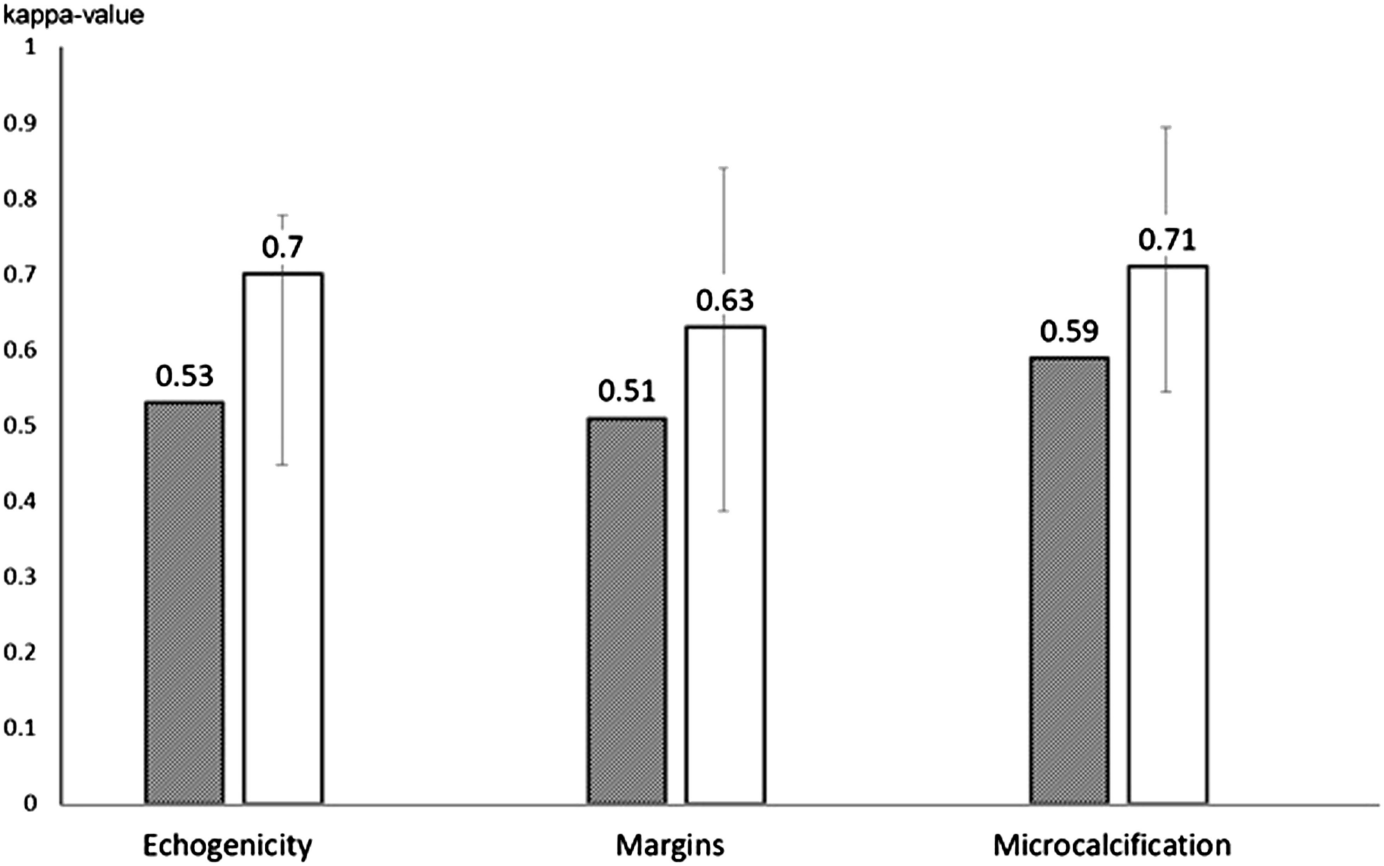
Intraobserver variation in the interpretation of certain ultrasound characteristics. For comparison purposes, we calculated Kappa-s, as earlier studies used Kappa values (31, 32, 42). Gray bars: present study; white bars: median of literature data. Error bars represent ranges.

Based on our findings, interobserver agreement was insufficient for the evaluation of nodule margins and moderate for microcalcifications, a clear difference compared to previous studies which found better agreement (19, 31, 32, 33, 34, 35, 36, 37, 38, 39, 40, 41, 42, 43, 44, 45, 46, 47, 48, 49) using still images (Fig. 2), while intraobserver variation was comparable (Fig. 3). This is explained almost exclusively by the difference in observer-dependent interpretation of nodule characteristics; while microcalcification and taller-than-wide shape have clear definitions, this is less true for nodule margins. Neither is there a well-defined reference for echogenicity; indeed, we have found poor interobserver agreement for the distinction between minimally/moderately and very hypoechoic nodules. On the other hand, good interobserver agreement has been found for the distinction between iso/hyperechoic and hypoechoic nodules, as well as for ETE.

Only ETE and presence/absence of a real nodule have a biological standard, namely pathology. However, significant interobserver variability is also described for the pathology assessment of ETE (50). Judgment of other characteristics relies on consensual interpretation of US images (7, 8, 9, 10, 11). The guidelines do not specify the number of protrusions or the extent that a protrusion must exceed in order to describe the margins as lobulated or spiculated. For nodule echogenicity, either the ‘normal thyroid’ or the strap muscles are used as reference. To increase confusion, it is unspecified if the muscle as a whole or only the muscle section with low adipose tissue content should be considered as reference tissue. The surrounding ‘reference’ thyroid tissue may be hypoechoic itself due to autoimmune thyroid disease or aging. Finally, there is a lack of clarity as to which combination of the three US features of ETE (discontinuous capsule, abutting, and bulging contours) offers the best combination of sensitivity and specificity for diagnosing ETE.

The size of the nodule was found to have a significant effect on interobserver variation. The larger the nodule the better the agreement for microcalcifications, while the smaller the nodule the better the agreement in discriminating between iso/hyperechogenic and hypoechogenic lesions. Nodule size was without effect on the evaluation of the borders of the nodule and ETE.

Our data on diagnostic sensitivity and specificity of microcalcifications, irregular margins, and nodule echogenicity are in agreement with those previously published in the literature (4, 6, 14, 15, 16, 17, 18, 19, 20, 21, 22, 23). Although three TIRADS scoring systems use US signs of ETE for predicting thyroid cancers irrespective of the real ETE (7, 8, 9), there is only one publication which evaluated the diagnostic sensitivity and specificity of US signs of ETE in this context (19). Similarly to Hoang and coworkers (19), we found that compared with other suspicious characteristics, US signs of ETE have a limited role in confirming malignancy while the lack of these signs provides excellent assurance for excluding malignancy.

The very high misclassification rate in ETE, when compared to histology, suggests that US might be a suitable tool for this purpose only in nodules ≤17 mm in maximal diameter. This raises a serious concern about the use of preoperative US for postoperative staging, as suggested by the current TNM classification (51).

Interobserver agreement for a given characteristic may be influenced by the number of choices offered to the examiner, especially if no universally accepted definition is available for each nodule characteristic, or the examiner is less trained. However, if there is one single ‘correct’ choice among the offered ones, and the examiner is in the possession of the widely accepted definition of the characteristics (choices), this effect ought to be less dependent on the number of choices offered and the ‘correct’ one easier selected. This adds support for the need of improving the consistency of the US lexicons and the terminology herein.

Interestingly, investigators described nodules in cases where histopathology failed to reveal a true nodule, and there was a good interobserver agreement in these cases. Disregarding the unlikely chance that the histopathologist missed a nodule, we conclude that there is an inherent weakness in that US, at no variance with any other imaging technique, may produce an identical visual image of a nodule in the absence of a true nodule. While we cannot offer a sound explanation of this, it is clearly worthy of further exploration.

Despite adequate power and surgical confirmation of all nodules studied, a limitation of our work is the relatively low number of patients. Moreover, taller-than-wide shape was not included in the analysis as a nodule characteristic. The reason being that we deemed it superfluous to test the US diameter measurement capability of expert US users. Two patients with thyroid malignancy other than thyroid cancer were also among the studied nodules, as by definition, consecutive cases were included. Strengths of our study include only evaluating surgically removed thyroid nodules, the US investigator team consisting of highly skilled physicians with extensive US experience, and the use of videos rather than still pictures thereby resembling the real-world situation. While the participation of experienced investigators might have positively affected sensitivity and specificity, the true extent and direction of this influence for the interpretation of our data remains unclarified and awaits testing in a number of different settings.

In conclusion, examination of video recordings, a condition close to the real-world situation, revealed substantial interobserver variation in the interpretation of each of the four important US characteristics of thyroid nodules. This variation was dependent on nodule size for microcalcifications and nodule echogenicity. The international establishment of uniformly accepted US definitions for nodule characteristics used by TIRADS is much needed. An international TIRADS accompanied by the development of a manual including an atlas of the images corresponding to standardization of the definitions for each sign used in TIRADS is warranted. When available, it remains to be proven whether teaching and implementing this instrument achieves a substantial improvement of the agreement in thyroid US reporting, and how this influences the use of FNA.

## Supplementary Material

Supplementary Table. Questions answered by the investigators for each nodule

Supplementary Material

## Declaration of interest

The authors declare that there is no conflict of interest that could be perceived as prejudicing the impartiality of the research reported.

## Funding

This study did not receive any specific grant from any funding agency in the public, commercial or not-for-profit sector.

## Author contribution statement

T S – study design, preparation of the 123 video records involved in the study, steering, first and last versions of the manuscript. L H – study design, steering, manuscript preparation, final manuscript approval. S B – evaluation of the cases, approval of the manuscript. A F – evaluation of the cases, approval of the manuscript. L J – evaluation of the cases, approval of the manuscript. G L K – evaluation of the cases, approval of the manuscript. E P – evaluation of the cases, manuscript preparation. K R – evaluation of cases, first version of the manuscript. G R – evaluation of cases, approval of the manuscript. Z K – development of the eCRF and the online evaluation system, statistical analysis. E V N – study design, steering, manuscript preparation, final manuscript approval.
