# Supplementary material for: Considerable interobserver variation calls for unambiguous definitions of thyroid nodule ultrasound characteristics
Source: Eur Thyroid J. 2023 Mar 15;12(2):e220134. doi: 10.1530/ETJ-22-0134 (PMC10083668; doi:10.1530/ETJ-22-0134)
Supplement: Supplementary Table. Questions answered by the investigators for each nodule [file supplementary_table_1.pdf]

Supplementary Table. Questions answered by the investigators for each nodule

1. Is the presented lesion a nodule? (Regardless of your choice here, please proceed with the evaluation.)
o Yes, it is
o No, it is not a nodule and I would not describe it as a nodule in my everyday practice
2. Comet-tail artifact
o Present
o Absent
o Uncertain
3. Figures in the back wall of cystic areas caused by posterior acoustic enhancement
o Present
o Absent
o Uncertain
4. Punctate echogenic foci (question based on ACR TI-RADS)
o Present
o Absent
o Uncertain but probably present
o Uncertain but probably absent
5. Unequivocal microcalcifications
o Present
o Absent
6. Hyperechogenic spots of uncertain origin
o Present
o Absent
7. Coarse calcification(s)
o Present
o Absent
o Uncertain
8. Central intranodular coarse calcification
o Present
o Absent
o Uncertain
9. Isolated macrocalcification occupying an entirely calcified nodule
o Present
o Absent
o Uncertain
10. Peripheral (rim) calcification(s)
o Present and disrupted with small extrusive soft tissue component
o Present and disrupted without small extrusive soft tissue component
o Present and not disrupted
o Absent
o Uncertain
11. Composition of the nodule
o Pure cyst
o Cystic or almost completely cystic
o Spongiform

○ Mixed cystic and solid
○ Solid or almost completely solid
○ Cannot be determined because of calcification(s)
12. Is the nodule partially cystic?
○ No
○ Yes, and the solid part is eccentric
○ Yes, and the solid part is not eccentric
13. Echogenicity
○ Hyperechoic or isoechoic 7
○ Mildly to moderately hypoechoic2
○ Very hypoechoic2
○ Anechoic2
○ Cannot be determined1
14. Nodule margin
○ Smooth
○ Ill-defined
○ Lobulated or irregular
○ Cannot be determined
15. Extrathyroidal extension
○ Present
○ Absent
16. Based merely on nodule ultrasound characteristics, would you recommend aspiration cytology?
○ Yes
○ No
17. Considering both the nodule characteristics and the provided (often limited) patient data, would you recommend aspiration cytology?
○ Yes
○ No
