# Supplementary material for: Considerable interobserver variation calls for unambiguous definitions of thyroid nodule ultrasound characteristics
Source: Eur Thyroid J. 2023 Mar 15;12(2):e220134. doi: 10.1530/ETJ-22-0134 (PMC10083668; doi:10.1530/ETJ-22-0134)
Supplement: Supplementary Material [file supplementary_material.pdf]

Supplementary Text 1

Protocol for the recorded ultrasound examinations

During ultrasound examination of the patients, first a quick scanning of one lobe was carried out in order to adjust the settings (depth, focus and frequency) to the characteristics of the given patient, if needed. Then, we performed transverse scanning, starting a bit lateral to the most lateral part of the thyroid, several centimeters above the thyroid lobe and downwards until reaching the clavicle. If required, we made a second and a third scanning closer to the midline. This was followed by a longitudinal scan starting laterally and proximal to the upper pole of the thyroid, followed by one or more longitudinal scans visualizing the lower parts of the lobe. This allowed measurement of diameters. Then, we focused on discrete lesions, which enabled judging specific ultrasound characteristics of the lesion. If a thyroid lobe was suspicious for extensive enlargement, we checked whether the lowest pole could be seen before and during swallowing. The narrowest and broadest diameter of the trachea was also measured at the level of the thyroid gland. The entire ultrasound examination was recorded.

For the purpose of the study, we edited 3 to 15 minutes of the examinations and prepared a representative clip. This always included at least one complete transverse and longitudinal scan, which started proximal/lateral to the nodule in question and terminated distal/medial to the nodule. The direction of the scanning was marked.
